# Pattern of care and treatment outcomes of metastatic non-clear cell kidney cancer: a single centre experience from India

**DOI:** 10.3332/ecancer.2024.1775

**Published:** 2024-09-23

**Authors:** Somnath Roy, Sreejata Raychaudhuri, Bivas Biswas, Deepak Dabkara, Arnab Bhattacherjee, Sandip Ganguly, Joydeep Ghosh, Yesha Sandipbhai Patel, Souhita Pal, Jagriti Karmakar, Anindita Mitra, Sujoy Gupta

**Affiliations:** 1 Department of Medical Oncology, Tata Medical Center, 14 MAR (EW), New Town, Rajarhat, Kolkata 700160, West Bengal, India; 2 Uro-Surgery, Tata Medical Center, 14 MAR (EW), New Town, Rajarhat, Kolkata 700160, West Bengal, India

**Keywords:** non-clear cell kidney cancer, demographic features, treatment response, outcomes

## Abstract

**Background:**

Non-clear-cell renal cell carcinoma (nccRCC) refers to a rare diverse heterogeneous group of tumours; usually treated with immune check point inhibitors and or tyrosine kinase inhibitors (TKIs). Prospective large-scale data from Asian countries is limited.

**Methods:**

This is a retrospective study of patients with metastatic nccRCC treated at Tata Medical Centre, Kolkata, India, from 2012 to 2022. Demographic profiles, histologic subtypes, treatment details, response to therapy (by response evaluation criteria in solid tumours (RECIST v1.1)) and survival status were captured from electronic medical records (EMRs) of hospitals up till May 2023. Kaplan Meier methods were estimated to assess progression-free survival (PFS) and overall survival (OS).

**Results:**

A total of 89 consecutive patients were screened for this study, 24 were excluded due to inadequate data in EMR. 65 patients were included in the final analysis, with a median age at diagnosis of 59 years (range 20–84) of which 81% were male. Histologic subtypes comprised of 43% papillary, 31% clear cell with mixed histology, 3% sarcomatoid and 23% others including chromophobe, mucinous-tubular, spindle cell, oncocytic, medullary, poorly differentiated and rhabdoid). The most common site of metastasis was the lung 62% (*n* = 40) followed by non-regional nodes 32%, bone 26% and liver 14%. 15% patients presented with haematuria and 62% underwent nephrectomy prior to systemic therapy. The majority received pazopanib 46% (*n* = 30), chemotherapy 20% (*n* = 13) including bevacizumab plus erlotinib, sunitinib 15% (*n* = 10) or cabozantinib 14% (*n* = 9). Only 3(5%) patients received nivolumab plus cabozantinib combination. Response to treatment showed complete response in 1.5%, partial response in 20%, stable disease in 51% and progressive disease in 23% as per RECIST v1.1. 17 patients required dose reduction and interruption due to adverse effects and 33% (*n* = 22) received second-line therapy with nivolumab 18% (*n* = 4), axitinib and everolimus among others. After a median follow up of 44 months, the median PFS was 13 months (95%CI 7.2–18.9) and the median OS was 17 months (95%CI 12.1–22.1) for the entire cohort.

**Conclusion:**

The overall response and survival for metastatic nccRCC was relatively better in comparison with published data, despite the limited number of patients treated with ICIs due to cost and access barriers.

## Introduction

Renal cell carcinoma (RCC) is one of the United States’ ten most common cancers. In 2023, the American Cancer Society reported that 81,800 new patients were diagnosed with cancers of the kidney and renal pelvis; it is currently one of the most frequently diagnosed cancers in men [1]. The incidence of RCC in Asia is lower, however, possibly due to underreporting [2].

There are two major histologic subtypes: approximately 75% of RCC is clear cell, and the remaining 25% is non-clear cell [3]. Non-clear cell renal cell carcinoma (nccRCC) is a heterogeneous entity that is further divided into several subtypes, such as papillary renal cell carcinoma (pRCC), chromophobe renal cell carcinoma (chRCC), collecting duct carcinoma, renal medullary carcinoma, translocation-associated renal cell carcinoma and unclassified renal cell carcinoma. Sarcomatoid differentiation can occur in both major subtypes of RCC in variable proportions [4].

At least 17 different genes that dysregulate tumour cells’ ability to respond to changes in oxygen, iron, nutrients and energy levels are associated with RCC [5]. The management of RCC has rapidly evolved with the introduction of drugs that can target these alterations in oxygen metabolism and cellular proliferation [6, 7]. Despite this, treating RCC remains particularly challenging because most RCCs are clinically silent and are diagnosed at an advanced or metastatic stage [8]. Novel therapies that focus on different downstream products of the von Hippel-Lindau/hypoxia-inducible factor pathway, such as the vascular endothelial growth factor (VEGF), transforming growth factor-α and platelet-derived growth factor-β, have demonstrated benefits in RCC treatment [9, 10]. Even certain subtypes of nccRCC like sarcomatoid differentiation usually resistant to traditional chemotherapy [11].

In recent years, promising clinical trial data has shifted the standard of care for advanced/metastatic RCC toward immune checkpoint inhibitors (ICIs), mostly with clear cell histology [12, 13]. Additionally, for nccRCC, there is limited data on the efficacy of other targeted therapies used in RCC, such as tyrosine kinase inhibitors (TKIs) and inhibitors of mammalian target of rapamycin (mTOR), so potential effects must be extrapolated from trials of ccRCC [14–16]. Currently, the National Comprehensive Cancer Network’s 2024 guidelines for managing advanced/metastatic nccRCC recommend enrollment in clinical trials or treatment with TKIs, such as cabozantinib, sunitinib or ICIs [17]. Prospective large-scale data from India on the management of nccRCC is limited [18–20]. Hence, we aimed to study the demographic profile and treatment outcomes of metastatic nccRCC patients from a tertiary care cancer center in India.

## Materials and methods

### Study population

This is a single-center, retrospective, non-interventional study on patients with metastatic nccRCC treated from May 2012 to December 2022 at Tata Medical Center, Kolkata, India. Consecutive patients who met the eligibility criteria were enrolled and followed up to May 2023. All patients aged ≥18 years registered in the Department of Medical Oncology with a histopathological diagnosis of RCC (stage IV, as per the American Joint Committee of Cancer Staging 6th and 7th edition) and non-clear cell histology were considered eligible for this study. Patients with measurable disease according to the response evaluation criteria in solid tumours (RECIST v1.1) were included in this study [21]. Patients with pure clear-cell histology were excluded. The survival analysis excluded patients with inadequate medical records and those who did not receive treatment after their initial evaluation. Due to the study’s retrospective nature, the investigators received a waiver of consent from the Institutional Review Board as per institutional policy (IEC Protocol Waiver no-EC/WV/TMC/09/23).

### Data collection and source

All data were obtained from hospital electronic medical records (EMRs). The demographic details included age, gender, presence of hematuria, sites of metastasis and Eastern Cooperative Oncology Group (ECOG) performance status. The histologic subtypes captured included papillary, sarcomatoid, clear cell with mixed histology and other variants. Treatment details, including for surgery (nephrectomy), first-line systemic therapy (including pazopanib, sunitinib, cabozantinib, chemotherapy plus minus bevacizumab and a combination of nivolumab plus cabozantinib) and second-line systemic therapy were retrieved. The response evaluation was performed using RECIST v1.1 criteria and dose modifications data were also captured. Dose reductions and dose interruptions are done according to standard guidelines based on the severity of adverse effects.

### Statistical analysis

There was no formal sample size calculation, as it was a single-institution, non-interventional, single-arm retrospective chart review. Descriptive statistics, tables and charts were used to analyze the demographic, clinical and treatment-related variables. The Kaplan–Meier method was used to estimate progression-free survival (PFS) and overall survival (OS). Patients who received at least 1 week of therapy were evaluated for survival outcomes and the data were censored on 31 May 2023. Patients who were lost to follow-up were censored at the time of last contact or follow-up. PFS was calculated from the date of initiation of systemic treatment to the date of disease progression or death from any cause. OS was calculated from the date of diagnosis to the date of death from any cause. Patients who were lost to follow-up or had abandoned therapy were included in PFS and OS analyses, and outcomes for these patients were confirmed via telephone contact. Statistical analysis was performed using STATA/SE 13.0 (StataCorp, College Station, TX).

## Results

A total of 89 consecutive patients were screened for this study. However, due to incomplete medical records in the EMR, 24 were excluded; as such, 65 patients were included in the final analysis. The median age at diagnosis was 59 years (range: 20–84) and 81% were male. The histologic subtypes comprised papillary (43%, *n* = 28), clear cell with mixed histology (31%, *n* = 20), sarcomatoid (3%, *n* = 2) and 23% (*n* = 15) other histologies, including chromophobe, mucinous-tubular, spindle cell, oncocytic, medullary, poorly differentiated and rhabdoid.

At the time of diagnosis, 15% of patients presented with hematuria. The most common site of metastasis was the lungs (62%, *n* = 40), followed by non-regional nodes (32%, *n* = 21), bones (26%, *n* = 17) and liver (14%, *n* = 9). The majority of patients (79%) had a good performance status of ECOG 0–1. A total of 62% (*n =* 40) underwent nephrectomy (either cytoreductive nephrectomy for localized disease or removal of a kidney tumor with palliative intent in a metastatic setting) before systemic therapy. The baseline demographics are shown in Table 1.

For first-line systemic therapy, the majority (46%, *n* = 30) received pazopanib, followed by chemotherapy (including bevacizumab) (20%, *n* = 13), sunitinib (15%, *n* = 10) and cabozantinib (14%, *n* = 9). Only 5% (*n =* 3) received nivolumab plus cabozantinib combination. For second-line therapy, 18% of patients received single-agent everolimus, nivolumab or axitinib and 14% of patients received combinations of either lenvatinib and everolimus or bevacizumab and erlotinib. Only 9% of patients were treated with either sorafenib or cabozantinib. One patient had a complete response to treatment, 20% (*n* = 13) had a partial response and 51% (*n* = 33), per RECIST v1.1 criteria, were classified as stable disease (SD). Seventeen patients (26%) required dose reduction and interruption due to adverse effects. Table 2 shows the treatment details and responses.

**Figure 1.**
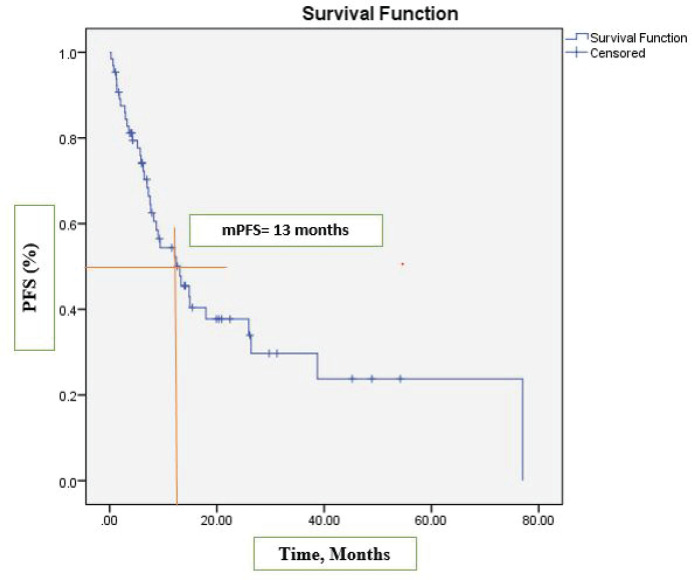
Kaplan-Meier curve for PFS.

**Figure 2.**
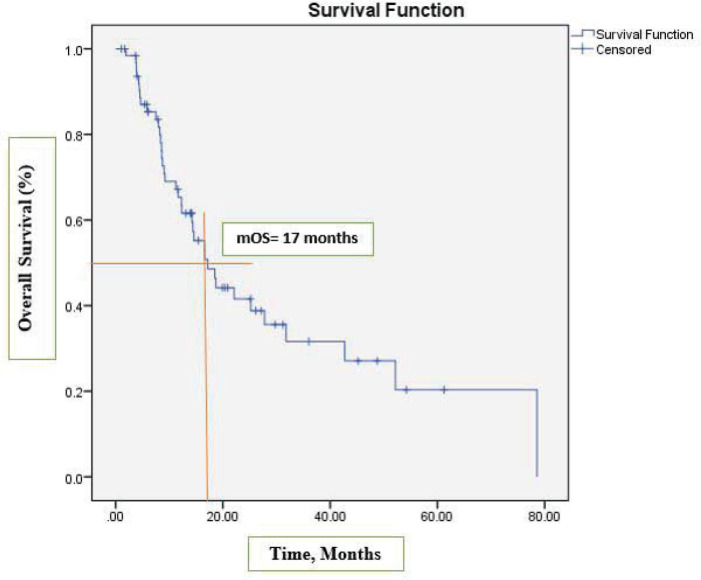
Kaplan-Meier curve for OS.

After a median follow-up at 44 months (95% CI, 39.2–54.3), the median PFS was 13 months (95% CI, 7.2–18.9) (Figure 1) and the median OS was 17 months (95% CI, 12.1–22.1). See Figure 2 for the entire cohort.

## Discussion

Non-clear cell histology is a less common variant of kidney cancer, so the optimal systemic therapy for advanced or metastatic disease remains still unclear. Most treatment recommendations have been extrapolated from clinical trials done on clear cell subtypes. Given the paucity of data, this retrospective study explored the demographic profile and treatment outcomes of metastatic nccRCC from the Indian cohort.

The modern therapeutic landscape and success in clear cell subtypes began in the early 2000s with the development of TKIs and mTOR inhibitors. This prompted investigators to examine responses to these agents in non-clear subtypes. Sunitinib, a VEGF-receptor TKI, demonstrated promising therapeutic activity in a multicenter phase II trial of 31 nccRCC patients (71% pRCC and 10% chRCC). Sunitinib had an objective response rate (ORR) of 35%, a median PFS of 6.4 months and a 1-year OS of 65% [22]. Subsequently, two randomized phase II trials conducted by the European Society of Parenteral and Enteral Nutrition (ESPEN) and the American Society of Parenteral and Enteral Nutrition (ASPEN) compared sunitinib with everolimus, an mTOR inhibitor. The ESPEN’s study, which included 68 patients, was the first head-to-head comparison of these two drugs and did not demonstrate a statistically significant difference in median PFS (6.1 versus 4.1 months) or OS (16.2 versus 14.9 months) [23]. The ASPEN’s research, which analyzed a larger cohort of patients (*n* = 108), found that sunitinib significantly increased the median PFS when compared to everolimus (8.3 versus 5.6 months) but did not significantly improve OS [24]. The Central European Society for Anticancer Drug Research trial compared sunitinib (*n* = 10) to another mTOR inhibitor, temsirolimus (*n* = 12) and reported a median PFS of 13.2 and 9.3 months and an OS of 19.8 and 19.4 months for sunitinib and temsirolimus, respectively [25].

Compared to sunitinib and pazopanib, axitinib has a higher affinity with VEGFR-1, VEGFR-2 and VEGFR-3 [26]. In a phase II study of predominantly pRCC patients (66% pRCC and 10% chRCC), pazopanib demonstrated promising therapeutic value with an ORR of 28%, a median PFS of 16.5 months and a 1-year OS of 69% [27]. Another phase II study demonstrated that axitinib was better when used after failure with temsirolimus in papillary and chromophobe subtype, with an ORR of 37.5%, median PFS of 7.4 months and median OS of 12.1 months [28]. To date, no trials have prospectively compared the efficacy of pazopanib and axitinib head-to-head in nccRCC, but the similar ORR and PFS data across trials highlights the utility of next-generation VEGFR TKIs in the non-clear subtype.

ICIs have also been tested, mainly in pRCC variants with apparent clinical activity. In KEYNOTE-427, a phase II study, enrolled 165 patients with nccRCC (pRCC (71.5%), cRCC (12.7%) and unclassified (15.8%)) and each received pembrolizumab 200 mg every 3weeks for ≤24 months with an ORR was 28.8% for pRCC, 9.5% for chromophobe [29]. In another phase, IIIb/IV CheckMate-374 trial of 44 patients with predominantly pRCC (*n* = 24); flat dose nivolumab 240 mg showed an ORR of 13% with a median OS of 16 months [30]. In phase II; savolitinib was combined with durvalumab with a response rate of 29% and median PFS of 4.9 months with a higher response with mesenchymal epithelial transition (MET) alteration in the papillary subtype [31, 32]. In another phase II trial of 47 patients with non-clear cell histology; nivolumab and cabozantinib showed an ORR of 47% with a median duration of response of 13.6 months and a PFS rate of 52% at 1 year [33]. The results of recent prospective clinical trials (S1500, PAPMET) suggest that cabozantinib should be the current first-line treatment for untreated pRCC [34]. In Keynote-B61, lenvatinib plus pembrolizumab showed promising results in non-clear cell subtypes [35].

Prospective data on the treatment of metastatic nccRCC from India is limited. Ramaswamy* et al* [18] conducted a registry-based retrospective study of 212 patients with metastatic RCC. In the study, 67.9% of patients had conventional clear-cell RCC and 32% had non-clear cell variants. TKIs (sunitinib, sorafenib and pazopanib) and mTOR inhibitors (everolimus and temsirolimus) were primarily used as the first-line treatment, with a median PFS of 7.09 months and median OS of 12.87 months for the entire cohort [18]. However, the study mainly included ccRCC cases, thus emphasizing the importance of our research, which focused solely on non-clear cell variants. The authors have acknowledged that the accessibility and affordability of such expensive targeted therapies limit the treatment of patients in resource-poor settings like India. Another retrospective study from a tertiary care hospital in Eastern India analyzed demographic and survival data for 81 patients with RCC [19]. The median OS for stage III was 52.9 months as compared with 18.7 months for metastatic RCC [19]. Agnihotri *et al* [20] observed 617 patients with renal tumors at a tertiary care center in Northern India; 67.7% had clear-cell subtype and 4.7% had metastatic disease. Younger patients had a higher proportion of non-clear cell RCC; only 48.6% had conventional ccRCC. Patients <39 years of age had a lower survival rate compared to those >70 years of age (5-year OS was 53% versus 80%, respectively) [20].

Our study’s strengths include the fact that to our knowledge, this is the first study focusing on 65 patients with non-clear cell RCC in a real-world Indian setting. The lack of proper treatment guidelines and paucity of large-scale prospective data; represent an unmet need that we attempted to address in this study with the best available resources. The survival analyses show encouraging results, which are better than published data despite financial and access barriers. The limitations of the study include the relatively small sample size and its retrospective nature. In addition, chemotherapy was given to some patients which is not the current standard. ICIs were not being able to offer due to availability and high cost. We acknowledge that we were unable to report toxicity data also.

## Conclusion

The overall response and survival of metastatic nccRCC were relatively better in this Indian cohort compared to previously published data, despite the limited number of patients treated with ICIs due to access barriers. However, gathering large-scale prospective data with proper treatment guidelines from the Indian context is an urgent need.

## Conflicts of interest

SR – received a PI grant in a clinical trial from AstraZeneca which was paid to the institution.

SG – received PI grant in a clinical trial from Novartis, AstraZeneca and Jhonson and Jhonson which was paid to the institution.

JG – received PI grant in a clinical trial from AstraZeneca which was paid to the institution.

BB – received PI grant in a clinical trial from Novartis, Pfizer, Roche, AstraZeneca, Jhonson and Jhonson which was paid to the institution.

AB – received PI grant in a clinical trial from AstraZeneca, Roche which was paid to the institution.

Other authors – ‘no conflicts of interest to declare.’

## Funding

No funding sources for this study.

## IRB approval

Taken (IEC Protocol Waiver no- EC/WV/TMC/09/23).

## Figures and Tables

**Table 1.** Demographic variables.

Variables	*n* = 65 (%)
Age (median)	59 (Range 20–84)
Gender Male Female	53 (81) 12 (18)
Haematuria Yes No	10 (15) 55 (85)
Histopathology types Papillary Sarcomatoid Clear cell with mixed histology Others (Mucinous-tubular, Spindle cell, Oncocytic, Medullary, Poorly differented, Rhabdoid, Chromophobe)	28 (43) 2 (3) 20 (31) 15 (23)
Sites of metastasis Lung Liver Bone Brain Distal nodes	40 (62) 9 (14) 17 (26) 1 (1.5) 21 (32)
ECOG PS 0 &1 2 & above	51 (79) 14 (21)
Nephrectomy Yes No	40 (62) 25 (38)

ECOG PS- Eastern Cooperative Oncology Group Performance Status

**Table 2.** Treatment details.

Variable	*n* = 65 (%)
First line therapy Pazopanib Sunitinib Cabozantinib Chemotherapy Nivolumab plus cabozantinib	30 (46) 10 (15) 9 (14) 13 (20) 3 (5)
Response Complete response Partial response SD Progressive diseases Unknown	1 (1.5) 13 (20) 33 (51) 15 (23) 3 (4.6)
Dose reduction and interruption Yes No	17 (26) 48 (74)
Second line therapy Everolimus Lenvatinib + Everolimus Nivolumab Axitinib Sorafenib Bevacizumab + Erlotinib Cabozantinib	4 (18) 3 (14) 4 (18) 4 (18) 2 (9) 3 (14) 2 (9)
